# Dataset on post-translational modifications proteome analysis of MSP1-overexpressing rice leaf proteins

**DOI:** 10.1016/j.dib.2023.109573

**Published:** 2023-09-13

**Authors:** Gi Hyun Lee, Cheol Woo Min, Jeong Woo Jang, Ravi Gupta, Sun Tae Kim

**Affiliations:** a Department of Plant Bioscience, Life and Industry Convergence Research Institute, Pusan National University, Miryang 50463, Republic of Korea; b College of General Education, Kookmin University, Seoul 02707, Republic of Korea

**Keywords:** Post-translational modifications, Rice blast disease, Plant-pathogen interaction, Proteomics

## Abstract

The data reported here are associated with the article entitled “Analysis of Post-Translational Modification Dynamics Unveiled Novel Insights into Rice Responses to MSP1” [1]. pathogen-associated molecular pattern (PAMP) -triggered immunity (PTI) serves as the fundamental defense mechanism in plants, providing innate protection against pathogen invasion. The fungus *Magnaporthe oryzae* (*M. oryzae*) secretes MSP1, a protein recognized as a PAMP that induces PTI responses in rice. However, the comprehensive characterization of MSP1-induced post-translational modifications (PTMs) and their contribution to PTI responses remains elusive thus far. In this manuscript, we report the analysis of the phosphoproteome, ubiquitinome, and acetylproteome to investigate the alterations in MSP1-induced changes in these PTMs in MSP1 overexpressed and wild-type rice, utilizing the QExactive^TM^ Orbitrap High-Resolution Mass Spectrometer [1]. Our data primarily focuses on unraveling the PTMs of MSP1-overexpressing transgenic rice, with the goal of elucidating MSP1-induced signaling cascades and deciphering their regulatory mechanisms.

Specifications Table

**Table:** 

Subject	Biology

Specific subject area	Plant science, Proteomics, Plant-pathogen interaction
Type of data	Tables, Figures
How the data were acquired	QExactive™ Orbitrap High-Resolution Mass Spectrometer (Thermo Fisher Scientific, USA) coupled with UHPLC Dionex UltiMate^TM^ 3000 (Thermo Fisher Scientific, USA) systems
Data format	Raw, Analyzed
Description of data collection	Rice leaves post-translational modifications proteome of wild type (cv. Dongjin, DJ) and extracellular MSP1 (eMSP1) overexpressed rice cultivars were analyzed
Data source location	Plant Immunity Laboratory, Department of Plant Science, Pusan National University, Miryang, Republic of Korea (latitude 35 N)
Data accessibility	Repository name: ProteomeXchange Data identification number: PXD039997. Direct FTP link to data: http://proteomecentral.proteomexchange.org/cgi/GetDataset?ID=PXD039997 Repository name: Mendeley Data Data identification number: DOI:10.17632/2z88ydwzzc.1 Title: Raw dataset for Integrated phosphoproteome, ubiquitinome, and acetylome analyses uncovering the post-translational modifications crosstalk during MSP1-induced signaling in rice Direct URL link to data: https://data.mendeley.com/datasets/2z88ydwzzc/1
Related research article	G.H. Lee, C.W. Min, J.W. Jang, Y. Wang, J.-S. Jeon, R. Gupta, Kim, S.T. Kim, Analysis of Post-Translational Modification Dynamics Unveiled Novel Insights into Rice Responses to MSP1 [1].

## 1 Value of the Data

• This dataset presents the identification of PTMs in rice leaf proteins induced by MSP1, a *M. oryzae* derived PAMP.
• Researchers in the field of plant-pathogen interactions can use our PTMs proteome data that contain the information of a total of 4666 modified sites in rice leaves, including 4292 phosphosites, 189 ubiquitin sites, and 185 acetylation sites, of which MSP1 significantly altered the PTM status of 437 phosphorylated, 53 ubiquitinated, and 68 acetylated peptides.
• The PTM datasets presented here can be valuable for future studies, allowing researchers to confirm PTMs associated with MSP1 or other PAMP molecules in rice using biochemical and molecular biology approaches, while also facilitating a deeper understanding of PTM-mediated signaling in plant-pathogen interactions.

## 2 Objective

This work aimed to elucidate the changes in PTMs of rice proteins induced by MSP1, a secreted PAMP from *M. oryzae*. In this manuscript, we present the comprehensive PTM proteomics analysis results, identifying the rice proteins from DJ and eMSP1 rice plants. The dataset encompasses a comprehensive analysis of PTMs, a total of 4666 modified sites in rice leaves, including 4292 phosphosites, 189 ubiquitin sites, and 185 acetylation sites. Notably, MSP1 induced significant alterations in the PTM status of 437 phosphorylated, 53 ubiquitinated, and 68 acetylated peptides among the identified rice proteins.

## 3 Data Description

**Fig. 1:**
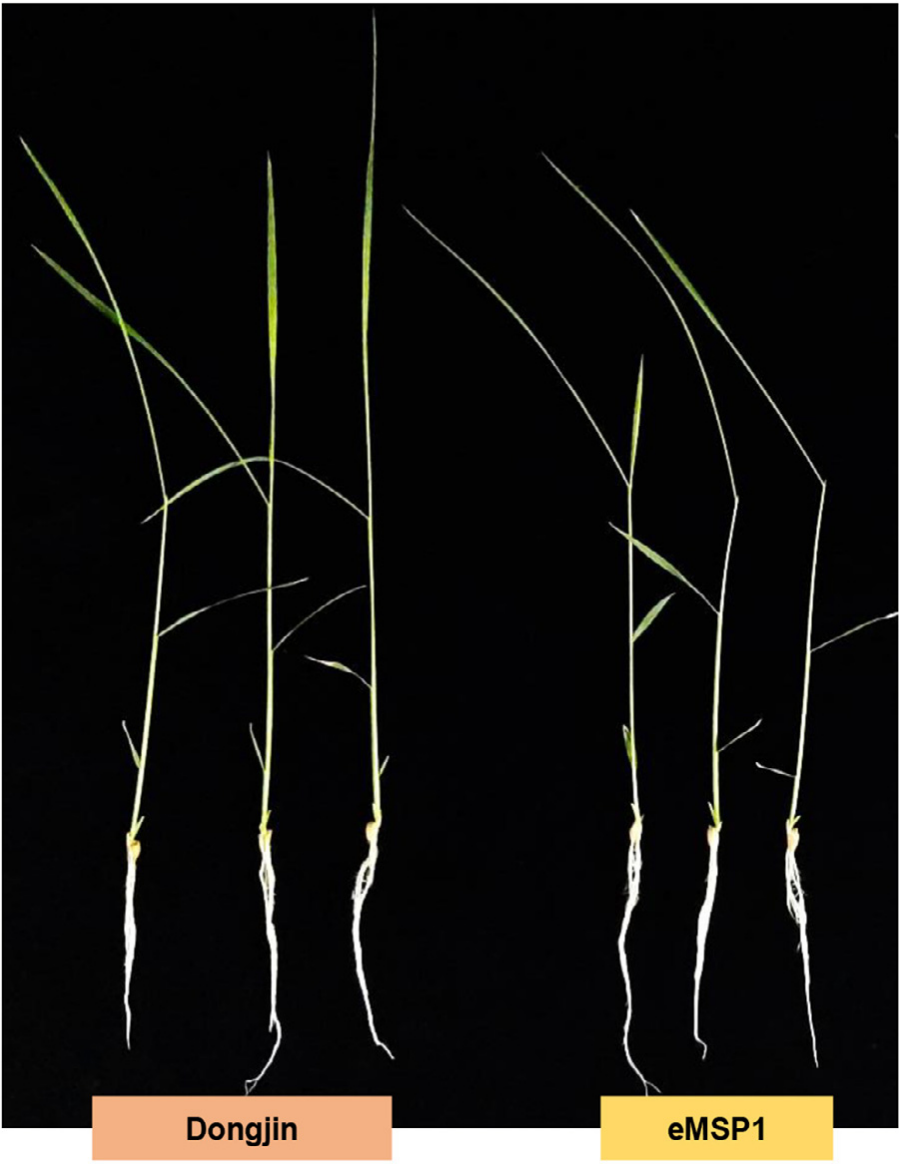
Rice seedlings of Dongjin, eMSP1 and cultivars 14 days after germination.

**Fig. 2:**
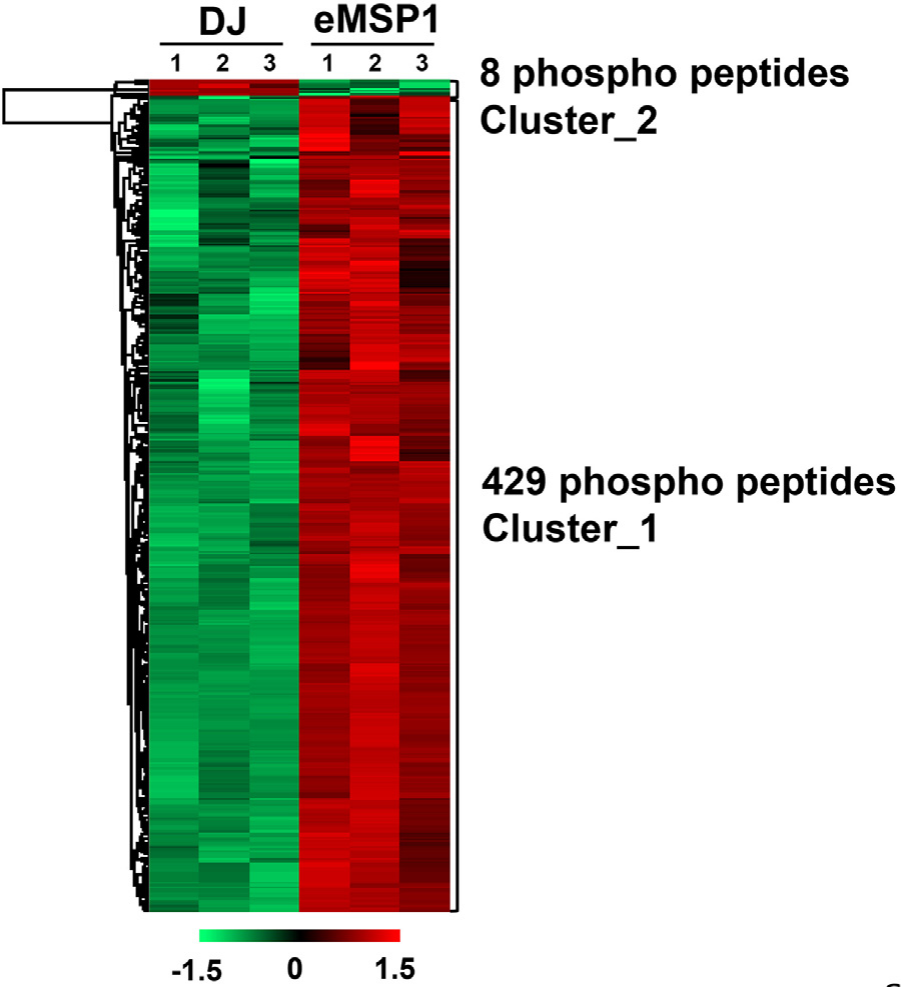
HCL analysis highlighting the relative fold change differences of phosphopeptides in response to MSP1.

**Fig. 3:**
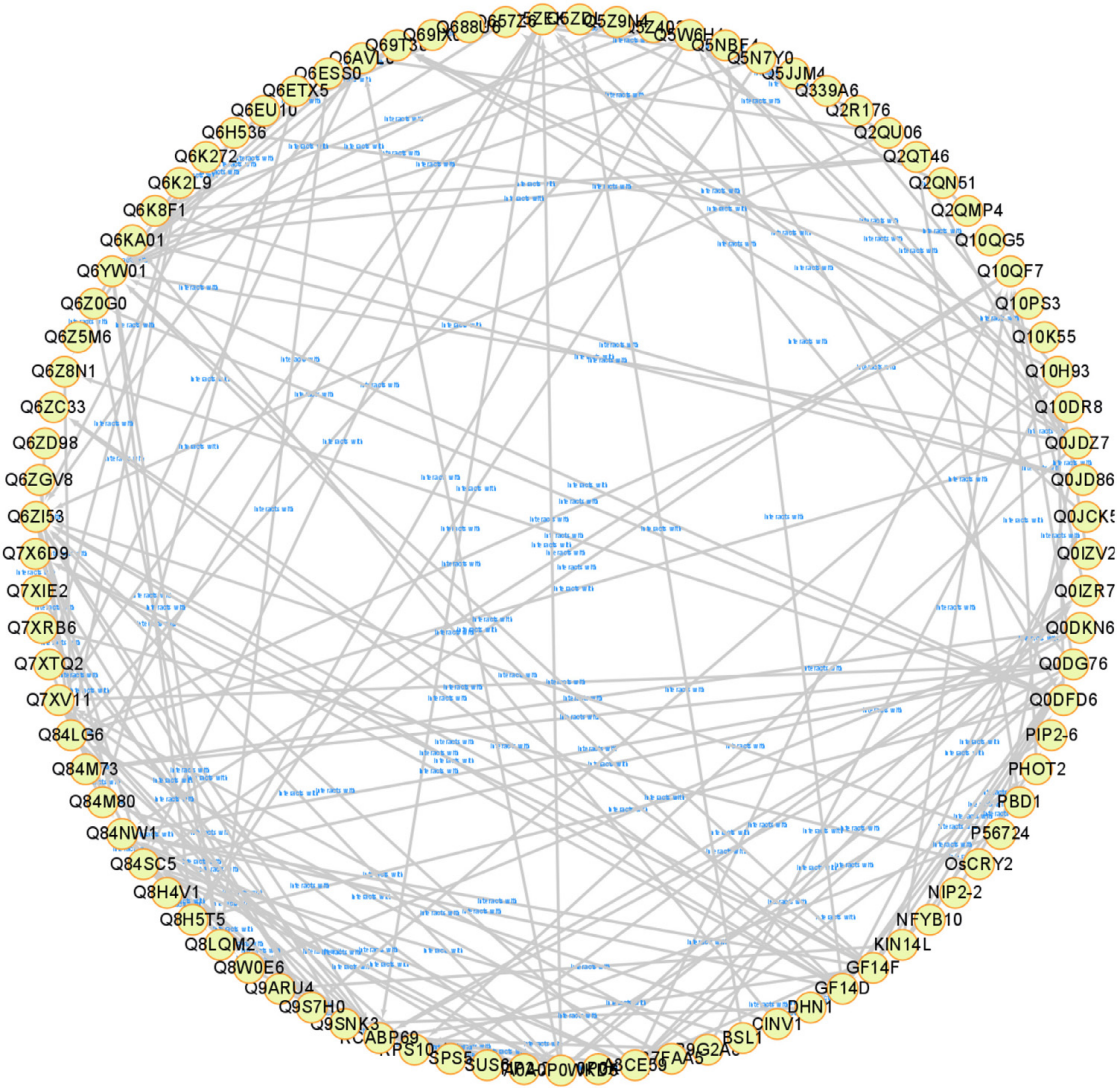
Interactome network of the identified phosphoproteins. Interaction network was constructed using STRING database and redesigned in the Cytoscape.

**Fig. 4:**
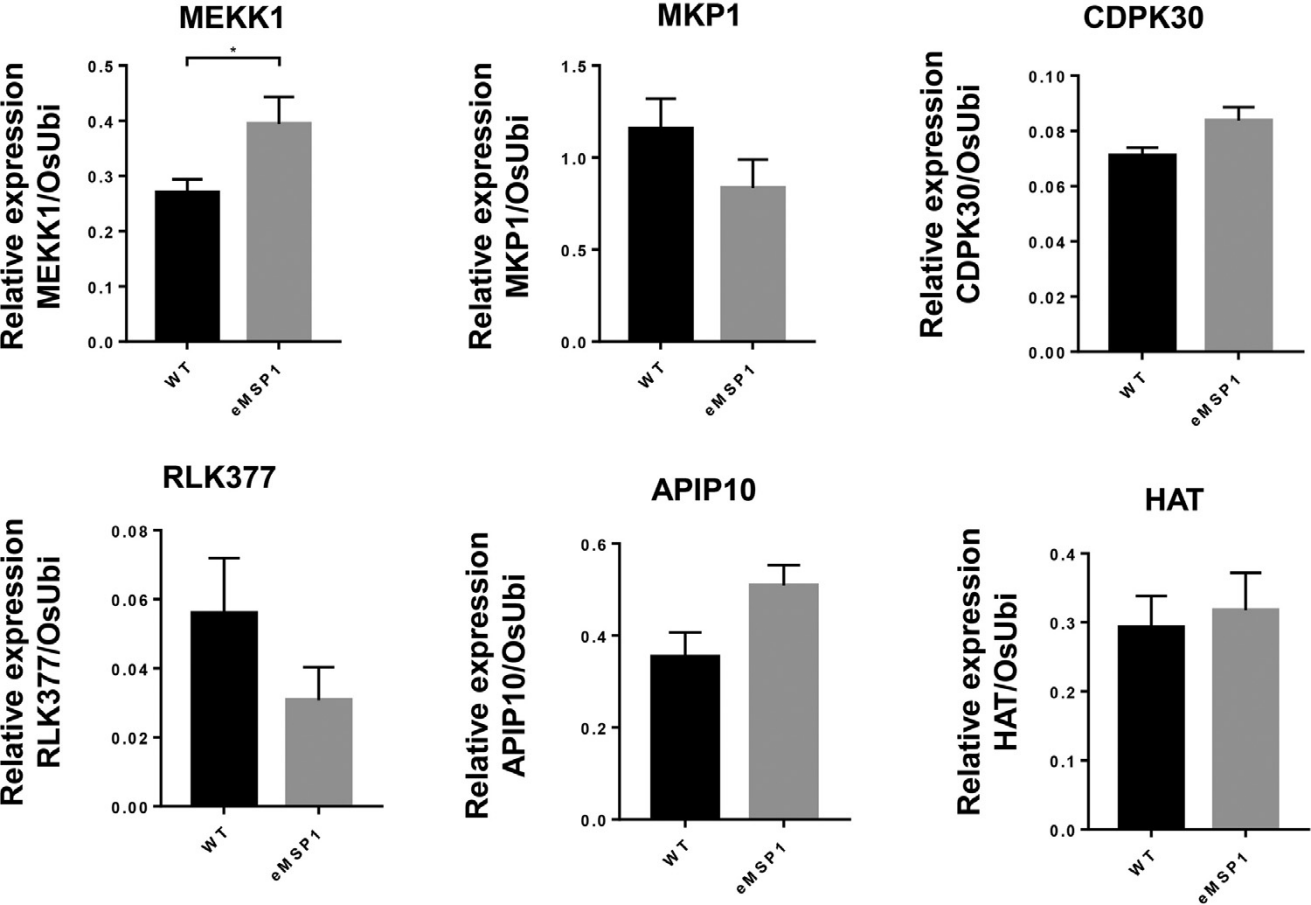
Bar charts showing the mRNA expression levels of MEKK1, MKP1, CDPK30, RLK377, APIP10, and HAT in WT and eMSP1 rice leaves by qRT-PCR analysis. Data are represented by the mean of at least three replicates, and error bars show the standard error (* *p* < 0.05 Student's t test).

**Fig. 5:**
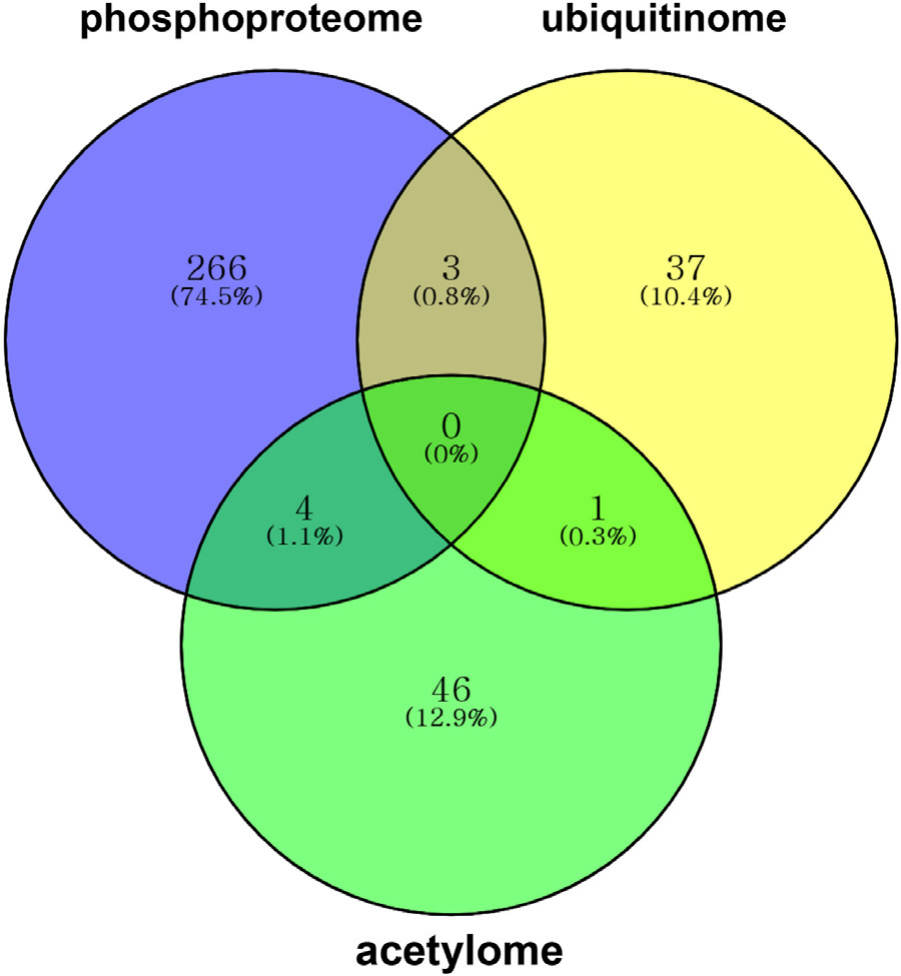
Venn diagram showing comparison and distribution of identified common proteins in phosphoproteome, ubiquitinome, and acetylome.

The dataset reported here was obtained from the PTMs analysis using DJ and eMSP1 overexpressed rice cultivars (Fig. 1). By employing a student's t-test with a *p*-value threshold of 0.05 and a fold change cut-off ≥1.5, 437 phosphopeptides were identified as significantly modulated, corresponding to a total of 274 phosphoproteins (Supplementary Table 1). Specifically, the hierarchical clustering analysis identified 429 phosphopeptides with increased abundance and 8 phosphopeptides with decreased abundance in the eMSP1 sample (Fig. 2). Subsequently, protein-protein interaction (PPI) network analysis was conducted utilizing the STRING web-based database to explore potential interactors associated with the identified phosphoproteins (Fig. 3). The application of the student's t-test, as described in the phosphoproteome analysis, was also extended to the analysis of the ubiquitome and acetylome. This analysis resulted in the identification of 53 significantly modulated ubiquitinated peptides and 41 ubiquitinated proteins (Supplementary Table 2). Furthermore, the acetylome analysis led to the identification of 68 significantly modulated peptides corresponding to 51 proteins (Supplementary Table 3). Among the PTMs-modified proteins identified in this study, the validation was focused on six key regulatory proteins associated with plant immune responses. These regulatory proteins included mitogen-activated protein kinase kinase kinase (MEKK1), MAPK phosphatase 1 (MKP1), Ca^2+^-dependent protein kinases 30 (CDPK30), receptor-like cytoplasmic kinase (RLCK377), AvrPiz-t interacting proteins 10 (APIP10), and histone acetyltransferase (HAT). RT-qPCR analysis was conducted to examine the corresponding mRNA levels of these key regulatory proteins (Fig. 4) and their corresponding primers are listed in Supplementary Table S4. To investigate the potential interplay among the identified PTMs in MSP1-induced signaling, we conducted a comparative analysis of the proteins modified by multiple PTMs. A total of three proteins were found to be common between the phosphoproteome and ubiquitinome. Additionally, we identified an overlap of four proteins between the phosphoproteome and acetylome and one protein that showed common modifications in both the ubiquitinome and acetylome (Fig. 5 and Supplementary Table 5).

## 4 Experimental Design, Materials, and Methods

### 4.1 Plant materials and growth conditions

Rice seeds of the *Oryza sativa* ssp. *japonica* cultivar Dongjin (DJ), including both the wild type (WT) and a transgenic line overexpressing MSP1 extracellularly (eMSP1), were subjected to overnight sterilization at 25 °C using a 0.05 % Spotak solution (Agrotech, South Korea), followed by five washes with distilled water. Germinated seeds were placed on moist tissue paper in Petri dishes and cultivated at 28 °C under a 16/8-hour light/dark cycle with 80 % humidity, using Yoshida solution. After four weeks, leaves from DJ and eMSP1 plants were quickly collected and stored at -70 °C to preserve protein quality and prevent degradation.

### 4.2 Total protein isolation and in-solution trypsin digestion for proteomic analysis

Total protein isolation from both wild-type (WT) and eMSP1 leaves was conducted following established methods [2,3]. A total 1g of finely-ground powder was homogenized with 5 mL of Tris-MG/NP-40 buffer [0.5 M Tris-HCl (pH 8.3), 2 % (v/v) NP-40, 20 mM MgCl_2_] and centrifuged at 14,000 × *g* for 10 min at 4 °C. The resulting supernatant was carefully transferred to a new tube and allowed to precipitate overnight at -20 °C using four volumes of TCA/acetone [12.5 % trichloroacetic acid (TCA) in 100 % acetone (w/v) containing 0.07 % (v/v) *β*-mercaptoethanol]. Pellets were obtained through centrifugation at 14,000 g for 10 min at 4 °C and subsequently washed three times with chilled 80 % acetone [80 % (v/v) acetone in distilled water containing 0.07 % (v/v) *β* -mercaptoethanol]. The washed pellet was dissolved in 80 % acetone and stored at -20 °C for further experiments.

In-solution trypsin digestion was performed utilizing the filter-aided sample preparation (FASP) method as previously described [2,3]. In brief, acetone-precipitated proteins were re-dissolved in denaturation buffer [4 % SDS and 100 mM DTT in 0.1 M tetraethylammonium tetrahydroborate (TEAB), pH 8.5] and quantified using the 2-D Quant kit (Cytiva, MA, USA). Subsequently, 400 µg of proteins were subjected to sonication for 3 min and heating at 99 °C for 30 min. The denatured proteins were then loaded onto a 30K spin filter (Merck Millipore, Darmstadt, Germany) and diluted with urea buffer [8 M urea in 100 mM TEAB, pH 8.5]. The protein-bound spin filter was washed three times with the urea buffer to remove SDS. Cysteine alkylation was achieved by incubating the proteins in alkylation buffer [50 mM iodoacetamide (IAA), 8 M urea in 0.1 M TEAB, pH 8.5] for 1 hour at room temperature, under dark conditions. Following alkylation, a trypsin solution (Promega, Madison, USA) dissolved in 50 mM TEAB containing 5 % acetonitrile (ACN) with an enzyme to substrate ratio [w/w] of 1:50 was added, and the proteins were digested overnight at 37 °C. The resulting digested peptides were collected through centrifugation and their total concentration was measured using the Pierce Quantitative Fluorometric Peptide Assay (Thermo Scientific, MA, USA), following the instructions provided with the kit.

### 4.3 Enrichment and purification of phosphorylated, ubiquitinated, and acetylated peptides

The enrichment of phosphopeptides was performed following a previously established study [2,3]. Lyophilized peptides were re-dissolved in the binding buffer provided in the TiO_2_ phosphopeptide enrichment kit (Thermo Scientific, MA, USA), followed by sonication and centrifugation. The TiO_2_ spin tip was equilibrated with buffer A [90 % ACN containing 0.4 % (v/v) Trifluoroacetic Acid (TFA)] and buffer B [25.65 % (v/v) of buffer A containing lactic acid]. The re-suspended peptide samples in 150 µL of buffer B were loaded onto the TiO_2_ spin tip and washed with buffer B and buffer A according to the manufacturer's protocol. Enriched phosphopeptides were subsequently eluted using elution buffer I [1.5 % ammonium hydroxide] and elution buffer II [2.5 % TFA]. Furthermore, modified peptides by ubiquitination and acetylation were sequentially enriched and purified using the PTMScan® ubiquitin remnant motif (K-ε-GG) kit and acetyl-lysine motif (Ac-K) kit (Cell Signaling, MA, USA), respectively. Lyophilized peptides were re-suspended in immunoaffinity purification (IAP) buffer, and the vial of the antibody-bead slurry was washed with phosphate-buffered saline (PBS) buffer. The antibody-bead slurry was then transferred to each sample and incubated on a rotator for 2 h at 4 °C. Subsequently, the samples were washed with ice-chilled ddH_2_O and eluted using elution buffer [0.15 % TFA]. The eluted peptides were lyophilized and stored at −70 °C until further LC-MS/MS analysis.

### 4.4 Q-Exactive MS analysis and raw data analysis

Peptides, dissolved in solvent-A (2 % ACN and 0.1 % formic acid), were separated using ultra-high-performance liquid chromatography (UHPLC) Dionex UltiMate^TM^ 3000 instruments (Thermo Fisher Scientific, MA, USA) [2,3]. The trapping of peptides was performed using an Acclaim PepMap 100 trap column (100 µm × 2 cm, nanoViper C18, 5 µm, 100 Å), followed by washing with 98 % solvent A. Peptide separation was achieved using an Acclaim PepMap 100 capillary column (75 µm × 15 cm, nanoViper C18, 3 µm, 100 Å) with a specific LC analytical gradient. The LC analytical gradient consisted of a gradual increase from 2 % to 35 % solvent-B (100 % ACN and 0.1 % formic acid) over 150 min, followed by a 10-min gradient from 40 % to 95 % solvent-B. The system then maintained 95 % solvent-B for 5 min before returning to 5 % solvent-B for 15 min. The electrospray ionization source was coupled to a quadrupole-based mass spectrometer (MS) QExactive™ Orbitrap high-resolution MS (Thermo Fisher Scientific, MA, USA) using the Top15 method for data acquisition. We employed an Ion Spray/nano spray ion source paired with an Orbitrap mass analyzer, covering a mass range of 40 to 6000 m/z. Scan speed exceeded 22 Hz, achieving a maximum mass resolution of 240,000 at a 1 Hz scan rate, with mass accuracy below 5 ppm RMS via external referencing.

The raw MS data were analyzed using MaxQuant (ver. 2.0.3.0) [4]. The rice protein samples were searched against the Phytozome *Oryza sativa* database (MSU v6.0, 67,393 entries) using the integrated Andromeda search engine [5]. Various modifications, including carbamidomethylation of cysteine residues, oxidation of methionine, acetylation of protein N-terminal, phosphorylation on serine, threonine, and tyrosine residues (phosphoSTY), ubiquitination of lysine residue (GlyGly(K)), and acetylation of lysine residues (Acetyl(K)), were considered during the search. A false discovery rate (FDR) of 1 % was applied for peptide identifications using a reverse nonsense version of the original database. Statistical analysis and evaluation of PTM datasets were performed using Perseus software (ver. 1.6.17.0) [6]. Missing values were imputed using a normal distribution method, and a two-sample t-test with a *p*-value threshold of 0.05 and a fold change greater than 1.5 was used to identify significant differences.

### 4.5 RNA extraction and gene expression analysis by RT-qPCR

RNA extraction was carried out from the rice leaves using TRIzol solution (Invitrogen, Carlsbad, U.S.A), followed by the synthesis of first-strand cDNA from 2 µg of the isolated RNA using M-MLV reverse transcriptase (Promega, Madison, USA). Real-time quantitative PCR (RT-qPCR) was conducted in triplicate using a Rotor-gene Q instrument (Qiagen, Hilden, Germany) and Prime Q-Mater Mix (Genetbio, South Korea), following the manufacturer's instructions. The specific primers utilized are listed in Supplementary Table S5. To determine the relative fold differences in template abundance for each sample, the threshold cycle (Ct) value for each gene of interest was normalized to the Ct value of OsUbi5 and calculated relative to a calibrator using the 2-ΔCT algorithm.

## Limitations

Not applicable.

## Ethics statements

No animal experiments were performed in this study.

## CRediT authorship contribution statement

**Gi Hyun Lee:** Software, Writing – original draft. **Cheol Woo Min:** Methodology, Software, Writing – original draft. **Jeong Woo Jang:** Methodology. **Ravi Gupta:** Methodology, Supervision, Writing – review & editing. **Sun Tae Kim:** Supervision, Funding acquisition, Writing – review & editing.

## Data Availability

The mass spectrometry proteomics data have been deposited to the ProteomeXchange Consortium via the PRIDE partner repository with the dataset identifier PXD039997.

## Appendix Supplementary materials

Image, application 1

Image, application 2

Image, application 3

Image, application 4

Image, application 5
